# Neuroanatomical Correlates of the Unity and Diversity Model of Executive Function in Young Adults

**DOI:** 10.3389/fnhum.2018.00283

**Published:** 2018-07-20

**Authors:** Harry R. Smolker, Naomi P. Friedman, John K. Hewitt, Marie T. Banich

**Affiliations:** ^1^Department of Psychology and Neuroscience, University of Colorado Boulder, Boulder, CO, United States; ^2^Institute for Behavioral Genetics, University of Colorado Boulder, Boulder, CO, United States

**Keywords:** executive control, neuroanatomy, individual differences, structural MRI, diffusion tensor imaging

## Abstract

Understanding the neuroanatomical correlates of individual differences in executive function (EF) is integral to a complete characterization of the neural systems supporting cognition. While studies have investigated EF-neuroanatomy relationships in adults, these studies often include samples with wide variation in age, which may mask relationships between neuroanatomy and EF specific to certain neurodevelopmental time points, and such studies often use unreliable single task measures of EF. Here we address both issues. First, we focused on a specific age at which the majority of neurodevelopmental changes are complete but at which age-related atrophy is not likely (*N* = 251; mean age of 28.71 years, *SD* = 0.57). Second, we assessed EF through multiple tasks, deriving three factors scores guided by the unity/diversity model of EF, which posits a common EF factor that influences all EF tasks, as well as an updating-specific and shifting-specific factor. We found that better common EF was associated with greater volume and surface area of regions in right middle frontal gyrus/frontal pole, right inferior temporal gyrus, as well as fractional anisotropy in portions of the right superior longitudinal fasciculus (rSLF) and the left anterior thalamic radiation. Better updating-specific ability was associated with greater cortical thickness of a cluster in left cuneus/precuneus, and reduced cortical thickness in regions of right superior frontal gyrus and right middle/superior temporal gyrus, but no aspects of white matter diffusion. In contrast, better shifting-specific ability was not associated with gray matter characteristics, but rather was associated with increased mean diffusivity and reduced radial diffusivity throughout much of the brain and reduced axial diffusivity in distinct clusters of the left superior longitudinal fasciculus, the corpus callosum, and the right optic radiation. These results demonstrate that associations between individual differences in EF ability and regional neuroanatomical properties occur not only within classic brain networks thought to support EF, but also in a variety of other regions and white matter tracts. These relationships appear to differ from observations made in emerging adults (Smolker et al., 2015), which might indicate that the brain systems associated with EF continue to experience behaviorally relevant maturational process beyond the early 20s.

## Introduction

Executive function (EF) is a set of domain-general cognitive control mechanisms supporting goal-directed behaviors (Banich, 2009). Lesion and functional neuroimaging studies have identified the prefrontal cortex (PFC) as being critically involved in supporting EF processes (for a review, see Alvarez and Emory, 2006), likely regulating behavior by biasing neuronal dynamics in more posterior brain regions supporting sensory processing, motor execution, and emotion, among other domains (Miller and Cohen, 2001; Banich, 2009; Depue et al., 2016). Specifically, functional magnetic resonance imaging (fMRI) studies evaluating the neural substrates associated with distinct EF dimensions have implicated regions of lateral PFC (Duncan and Owen, 2000; Wager and Smith, 2003; Collette et al., 2005; Petrides, 2005; Banich, 2009) and medial PFC (Wager et al., 2004; Derrfuss et al., 2005) in supporting discrete EF constructs analogous to those evaluated in the current study. Nonetheless, it remains unclear whether *individual differences* in EF abilities in healthy individuals who do not suffer from neurological insult are associated with *neuroanatomical* characteristics of these same PFC brain regions. An alternative possibility, which we explore in the current study, is that higher levels of EF are associated with regions outside of the PFC, and potentially even outside of the fronto-parietal network (FPN). Such findings would suggest that higher levels of EF are characterized by the potential for larger participation and/or distribution of processing across the brain.

In the current study, we focus on neuroanatomical correlates of EF derived from structural MRI (sMRI) including surface based morphometry (SBM) and diffusion-tensor imaging (DTI). Unlike fMRI, which is fleeting and susceptible to confounding factors such as fatigue (Peltier et al., 2005; Cook et al., 2007), stimulus exposure (Grill-Spector et al., 2006), and mood (Posse et al., 2003), sMRI provides relatively stable metrics of brain organization that may change with development, but do not fluctuate on a day-to-day or moment-to-moment basis, as fMRI may. Of particular interest is the degree to which individual differences in neuroanatomy – specifically gray matter volume, thickness, surface area, and local gyrification, as well as white matter diffusion properties, including fractional anisotropy, mean diffusivity, axial diffusivity, and radial diffusivity, are associated with individual differences in EF ability.

To date, research into the neuroanatomical correlates of EF abilities have largely implicated regions within the FPN, specifically the PFC, as being central to EF task performance (Gunning-Dixon and Raz, 2003; Van Petten et al., 2004; Zimmerman et al., 2006; Vasic et al., 2008; Depue et al., 2010; Salthouse, 2011; Yuan and Raz, 2014; Bettcher et al., 2016). However, most research on associations between neuroanatomy and individual differences in EF in healthy adults have had two characteristics that make drawing conclusions somewhat difficult. First, much of the existing research on the neuroanatomical correlates of EF fail to differentiate between EF and non-EF processes that contribute to task performance on any given EF task, and thus suffer from the “task impurity problem” (Miyake et al., 2000). Second, many studies have employed samples that span a wide range of ages (Zimmerman et al., 2006; Newman et al., 2007), including individuals for whom the brain is continuing to develop (i.e., individuals in their teens and early 20s) as well as individuals for which atrophy may have already commenced, such as those middle-aged and beyond (Zimmerman et al., 2006; Newman et al., 2007). The current study addresses both of these issues. We characterize each individual’s EF performance across a battery of tasks, allowing us to compute EF factor scores that provide a measure of an individual’s EF abilities less contaminated by specific requirements for any given task. In particular, we derive three EF factors dimensions as posited by the unity/diversity model of EF, a well validated individual differences model of EF (Friedman et al., 2008; Miyake and Friedman, 2012; Friedman and Miyake, 2017). The association of these factor scores with neuroanatomical characteristics of the brain are then investigated in a developmentally homogenous sample of adults (all within a year of 29 years of age), following up on our prior investigations with an emerging adult (i.e., college-aged) sample (Smolker et al., 2015). We discuss each of these issues in turn.

A major challenge to a clear understanding of the relationship between individual differences in EF and brain anatomy is that there is very little agreement across studies as to the best models and methodologies to characterize EF. One issue is that performance on any given EF task likely taps both EF and non-EF processes (Denckla, 1996; Rabbitt, 1997; Miyake et al., 2000), such as speed of processing, visual acuity, amongst others. To reduce this “task impurity problem,” EF researchers have used latent variables or composites of performance across tasks that tap the same EF, each of which require different non-EF processes (Miyake et al., 2000). While these factor analytic methodologies have been frequently employed in general research on EF, such methodologies have been scarcely employed in trying to understand the neuroanatomical correlates of discrete EF constructs. Although researchers have tested models of EF that posit multiple EF constructs (Robbins, 1996; Stuss and Alexander, 2007; Stuss, 2011), studies evaluating the *neuroanatomical correlates* of EF have rarely measured individual differences on EF constructs through factor analyses across multiple, reliable EF tasks. As such, different studies are investigating different “slices” of EF, making it difficult to interpret across studies (i.e., whether results are not replicating, or whether individual differences in distinct aspects of EF are associated with distinct neuroanatomical substrates). In past (Smolker et al., 2015), present, and future studies, our research team is attempting to conduct a series of studies examining the linkage between individual differences in EF and that employs consistent methodologies so as to allow for meaningful comparisons across samples, such as those in different age segments across the lifespan.

An abundance of evidence suggests that there are multiple separable constructs at the core of EF ability (Alvarez and Emory, 2006; Stuss and Alexander, 2007; Friedman and Miyake, 2017). Although EF has been operationalized under a number of frameworks (Baddeley, 1996; Miyake et al., 2000; Banich, 2009), the unity/diversity model of EF has emerged as a powerful model for interrogating the mechanistic structure of EF in the context of individual differences (Friedman and Miyake, 2017). With confirmatory factor analysis, the unity/diversity model partitions performance on multiple EF tasks into separable EF dimensions, and also reduces measurement error, providing a more accurate estimate of an individual’s underlying EF abilities (Miyake et al., 2000). Specifically, the model captures the correlations among response inhibition, working memory updating, and mental set shifting tasks with three orthogonal factors: a common factor that is involved in all EF tasks, known as common EF, as well as two separable and more specific factors, known as shifting-specific EF and updating-specific EF, respectively. Common EF captures variance in performance that is shared between EF tasks, and has been conceptualized as an ability to maintain a task set or goals. Shifting-specific and updating-specific represent residual covariance among mental set shifting tasks and working memory updating tasks, respectively, after variance due to common EF has been removed. Shifting-specific is thought to reflect the speed with which no-longer-relevant goals can be cleared from working memory, while updating-specific is thought to reflect the accuracy of working memory gating and possibly retrieval processes (Miyake and Friedman, 2012; Friedman and Miyake, 2017). There is no inhibition-specific factor, because once the common EF factor is in the model, there are no remaining correlations among the inhibition tasks; that is, common EF captures all the individual differences in response inhibition (see Friedman and Miyake, 2017, for further discussion).

Studies with adult samples spanning wide age ranges or with clinical populations generally demonstrate that impairment in EF abilities is associated with reductions in neuroanatomical properties within regions of the FPN, including measures of gray matter morphometry and diffusion characteristics of white matter. Of note, however, not only do such studies generally fail to use specific models of individual differences in EF, like the unity/diversity model, many of the studies that have investigated the relationship between level of EF ability and neuroanatomy have done so in the elderly (Gunning-Dixon and Raz, 2003; Van Petten et al., 2004; Kramer et al., 2007; Salthouse, 2011; Bettcher et al., 2016) or across a wide expanse of age, ranging from the teens to 60s or 70s (Zimmerman et al., 2006; Newman et al., 2007). Recent research suggests that the late teens and early 20s are an especially active times in brain development (Gogtay et al., 2004), a developmental time period that some have referred to as “emerging adulthood” (e.g., Arnett, 2000). It is becoming increasingly clear that during this time period, aspects of brain morphology and white matter diffusion continue to develop (Sowell et al., 2001, 2003; Mukherjee et al., 2002; Asato et al., 2010), with levels of multiple neuroanatomical properties not reaching stability until around 30 years of age, if not older (Sowell et al., 2003; Westlye et al., 2009). Conversely, aspects of brain atrophy can start to be observed (Rusinek et al., 2003; Salthouse, 2011; Bettcher et al., 2016) in the 60s and 70s. As such, employing samples that are heterogeneous for age and/or developmental status may obscure informative brain-behavior relationships that are only present during specific developmental periods. This presents a challenge to traditional practices used to investigate individual differences, in which maximum between-subject variability in dependent and independent variables is desirable. Whereas many studies of individual differences attempt to increase between-subject variance to have a better chance at detecting an effect, in the current study, we sought to minimize between-subject variance attributable to age.

Studies that have examined the relationship between neuroanatomical structure and individuals suffering from psychopathology (Szeszko et al., 2000; Pantelis et al., 2002; Makris et al., 2006; Rüsch et al., 2007; Watari et al., 2008; Keller et al., 2009; Depue et al., 2010) also provide limited information regarding individual differences in EF and brain structure in the neurologically normal brain, as these are populations in whom brain structure is likely altered. Findings within neurologically normal individuals regarding the relationship of individual differences in EF and brain neuroanatomy have been highly inconsistent. Whereas some studies report positive correlations between level of EF and aspects of brain neuroanatomy (i.e., better EF associated with greater neuroanatomy; Ettinger et al., 2005; Newman et al., 2007; Elderkin-Thompson et al., 2008; Gautam et al., 2011), others report negative (Gautam et al., 2009, 2011; Tamnes et al., 2010; Smolker et al., 2015) relationships across the brain (i.e., better EF associated with reduced neuroanatomy). As such, the degree to which the neuroanatomy of PFC regions predicts levels of EF in non-aging or non-clinical populations remains to be seen. To address this, we focus our investigation on individuals in a relatively limited but developmentally stable time frame of what we refer to young adulthood, around the age of 30.

A very limited number of studies have examined the neuroanatomical correlates of EF from the perspective of the unity/diversity model, employing factor analyses across multiple tasks, in neurologically normal populations. These investigations have been limited so far to a sample of children and adolescents (Tamnes et al., 2010), as well as emerging adults (Smolker et al., 2015). None have done so in a population in whom most major neurodevelopmental processes are complete. Tamnes et al. (2010) found that, across childhood and adolescence, improved performance on tasks tapping three correlated EF dimensions (response inhibition, working memory updating, and set shifting), was associated with reductions in cortical thickness across a number of brain regions. Specifically, better performance on the antisaccade task, a proxy for common EF, was associated reductions in cortical thickness of bilateral occipital lobe. Better performance on the keep track task, a proxy for updating (which contains variance related to common EF and updating-specific), was associated with reductions in cortical thickness of a portion of left dlPFC, extending back into bilateral postcentral gyrus (Tamnes et al., 2010). Finally, better performance on the plus-minus task, a proxy for shifting-shifting (which contains variance related to common EF and shifting-specific), was associated with reductions in cortical thickness of left precentral gyrus. Of these regions, only the dlPFC region associated with updating is considered part of the FPN (for a characterization of the FPN, see Yeo et al., 2011), suggesting that individual differences in EFs are associated with neuroanatomy both within and outside of the FPN, at least in childhood and adolescence.

Similarly, our research group found that each of the different dimensions of the unity/diversity model were associated with different aspects of brain neuroanatomy in a sample of emerging adults tightly centered around college age and somewhat overlapping in age with Tamnes and colleagues’ sample (Smolker et al., 2015). Specifically, we found that less regional gray matter volume and local gyrification within the PFC, as well as increased fractional anisotropy of white matter tracts (a measure of white matter integrity), connecting the PFC to posterior brain regions, were associated with higher EF ability. Specifically, better common EF was associated with reduced volume and local gyrification of ventromedial PFC and greater fractional anisotropy of the right superior longitudinal fasciculus. Moreover, better updating-specific was associated with decreased volume and gyrification of left dorsolateral PFC (dlPFC), and better shifting-specific was associated with reduced volume and local gyrification of the right ventrolateral PFC and increased fractional anisotropy of the right inferior fronto-occipital fasciculus. Of these results, as was the case in Tamnes et al. (2010), the only association between EF and regional neuroanatomy within the FPN was between updating-specific and dlPFC neuroanatomy, in this case with regards to volume and gyrification. The common EF and shifting-specific associations, though observed within the PFC, were not in regions commonly considered as being part of the FPN.

Taken together, these two studies provide converging evidence that, across childhood, adolescence, and early adulthood, (1) the unity/diversity dimensions of EF (or related tasks/constructs) are associated with neuroanatomy both within and outside of the FPN, and (2) there might be heterogeneity across age groups in the neuroanatomical regions associated with EF. However, it is difficult to disentangle age effects from effects driven by methodological differences between studies. On the one hand, in both Tamnes et al.’s (2010) child/adolescent and Smolker et al.’s (2015) emerging adult samples, updating performance was negatively correlated with dlPFC neuroanatomy. On the other hand, the regions associated with common EF and set shifting appeared to differ between age groups. An additional commonality between the results of Tamnes et al. (2010) and our research group (Smolker et al., 2015) is that reduced gray matter morphometry was associated with better EF performance. While at first glance this finding may seem counterintuitive, the brain is undergoing significant pruning during these ages, through which superfluous neurons are culled and the brain undergoes regional gray matter shrinkage, resulting in increased neural efficiency (Blakemore and Choudhury, 2006). Taken together, these results paint a picture of childhood through young adulthood in which better EF might be associated with individual differences in pruning of specific brain regions, with those individuals who have experienced greater pruning (and thus reduced gray matter morphometry) having better EF ability. At least in young adults, these negative associations between gray matter morphometry and EFs coincide with associations between white matter properties and EF, which may partially mediate gray matter-EF relationships (Smolker et al., 2015).

Given that the brain is likely still undergoing major developmental changes during the age ranges examined in both of the aforementioned samples, the negative correlations between gray matter morphometry and the unity/diversity dimensions may not be indicative of the relationships that would be observed in young adults. Indeed, studies evaluating neuroanatomical changes across the lifespan find that individuals in their early 20s are still undergoing neuronal pruning and axonal myelination, which may persist throughout much of the 20s. These effects stabilize around 30 years of age, and begin to change again around age 60, as individuals start to experience age-related neurodegeneration (Sowell et al., 2003; Westlye et al., 2009). As such, negative correlations between EF performance and gray matter morphometry during adolescence/emerging adulthood may exist because individuals with greater pruning are likely further along in typical neurodevelopmental processes, resulting in a) better EF and b) reduced gray matter morphometry. It remains unclear, however, whether the same neuroanatomical properties that are associated with EF during ages at which pruning and myelination is ongoing will also be associated with EF in individuals for which pruning and myelination has largely finished. This uncertainty applies not only to the specific neuroanatomical properties implicated between different age groups but also to the regions implicated. For instance, it may be that neuroanatomical properties of the PFC are particularly relevant to individual differences in EF during development, but as individual’s complete development, the variability in properties of the PFC between subjects becomes minimal and properties of the PFC are no longer relevant to individual differences in EF. Instead, the specific neuroanatomical properties and brain regions associated with EF may change across the lifespan, with distinct neuroanatomical correlates of EF occurring at distinct points in the lifespan.

Hence, in the present study, we focused on an age range in which the vast majority of neurodevelopmental processes associated with adolescence and emerging adulthood are likely to be over, but one at which age-related cognitive decline (and potential brain atrophy) are not likely yet to manifest. In a sample whose age is tightly focused around 30, we test for associations between individual differences in EFs and regional brain neuroanatomy, including characteristics of gray matter morphometry (volume, thickness, surface area, and local gyrification index), as well as multiple measures of white matter diffusion (fractional anisotropy, mean diffusivity, radial diffusivity, axial diffusivity). In addition to testing for gray matter morphometry and DTI measures on a whole-brain basis, we also employed ROI analyses based on gray matter regions and white matter tracts associated with EFs in emerging adults (Smolker et al., 2015). Consistent with Smolker et al. (2015), we expected that regions of gray matter and white matter tracts associated with individual differences in EF will not be restricted to the FPN, but will likely include other prefrontal and posterior brain regions outside of the FPN. Due to developmental differences between the current sample and the sample employed in Smolker et al. (2015), we expect that the direction of the relationship between measures of gray matter morphometry, DTI measures, and EF may differ and/or the regions implicated as related to individual difference in EFs may also be distinct.

## Materials and Methods

### Participants

Participants were 251 individuals drawn from the larger Colorado Longitudinal Twin Study (LTS) who were scanned when they were mean age 28.71 years (*SD* = 0.57). Of these 251 individuals, 108 were monozygotic (MZ) (72 female), 88 were dizygotic (DZ) same-gender (54 female), and 55 were singletons (28 female) whose co-twins had not participated at the time of the analyses. Written informed consent was obtained from all participants prior to carrying out the experimental session. This study was carried out in accordance with the recommendations of University of Colorado Boulder Institutional Review Board with written informed consent from all subjects. All subjects gave written informed consent in accordance with the Declaration of Helsinki. The protocol was approved by the University of Colorado Boulder Institutional Review Board prior to data collection. Structural images for neuroanatomical analyses were collected as part of a larger protocol including fMRI scans during tasks and at rest.

### EF Measures

The three EF constructs posited by the unity/diversity model of EF were assessed with six tasks previously shown to load on these constructs (Friedman et al., 2016). These six tasks were selected based on their factor loadings from prior waves of assessment using nine EF tasks in this sample (Friedman et al., 2016). The antisaccade, category-switch, and keep track tasks were completed during an fMRI session immediately following the T1 structural scan. Participants practiced these tasks outside the scanner prior to the scanning session to ensure they understood the tasks. They were reminded of the instructions at the beginning of the scanner tasks. The Stroop, letter memory, and number–letter tasks were completed as part of a larger behavioral battery immediately after the scanning session.

#### Antisaccade [Adapted From Roberts et al. (1994)]

Antisaccade captures the ability to maintain and execute a task set in the face of distracting information; specifically, it requires inhibiting prepotent eye movements (Miyake et al., 2000). In the scanner version, participants completed 20 s blocks of prosaccade, antisaccade, and rest (fixation) trials (12 blocks of each across two runs; 5 trials per block for the prosaccade and antisaccade blocks), each preceded by a jittered instruction (TOWARD, AWAY, or FIXATION for 2, 4, or 6 s). On each trial, after a jittered fixation lasting 1–3 s, a small visual cue flashed on one side of the computer screen for 234 ms, followed by a target (a digit from 0 to 9) that appeared for 150 ms before being masked. The mask lasted 1650 ms, during which time the participant vocalized the target. The cue and target appeared on the same side of the screen during prosaccade trials and opposite sides during antisaccade trials. Thus, to see the target for long enough to identify the number in the antisaccade trials, participants had to avoid the automatic tendency to saccade to the cue and instead immediately look in the opposite direction. The dependent measure was the proportion of correctly identified targets on the 60 antisaccade trials.

#### Stroop [Adapted From Stroop (1935)]

Stroop captures the ability to maintain a task set in the face of the distracting information, specifically, inhibiting the prepotent tendency to read words. Participants verbally indicated the font color (red, blue, or green) of text presented on a black screen as quickly as possible, with reaction time (RT) measured via a ms-accurate voice key. Trials were divided up into three types: a block of 42 neutral trials consisting of asterisks (3–5 characters long) presented in one of three colors; a block of 42 congruent trials consisting of color words that matched the font color (e.g., the word “RED” displayed in red font); and two blocks of 42 trials each of incongruent trials consisting of color words that did not match the font color (e.g., the word “RED” displayed in blue ink). Each word disappeared as soon as the voice key detected the response, and the next word appeared after a 250 ms white fixation. The dependent measure was the mean RT difference between correct incongruent and neutral trials.

#### Keep Track [Adapted From Yntema (1963)]

Keep track captures the ability to maintain and update information in working memory. On each trial in the scanner version, participants were given 3 or 4 target categories (animals, colors, countries, distances, metals, or relatives) that remained on the screen throughout the trial. After viewing a serial list of 16 words drawn from 6 categories (one word every 2 s), they saw a “???” prompt on the screen for 10 s, during which they orally recalled the last exemplar of each target category. Because each list contained 1–3 exemplars of each category, they had to update which words to remember and ignore words from irrelevant categories. In addition to these “Remember” trials, the scanner version of the task included baseline conditions of “Read” trials, in which participants just silently read the words without trying to remember them, and 20 s rest (fixation) trials. Each trial type was preceded by a jittered instruction (REMEMBER, READ, or FIXATION for 2, 4, or 6 s). There were three runs, each with 3 recall trials (two with 4 words to recall and one with 3), 3 read trials, and 3 rest trials. The behavioral dependent measure was the proportion of the 33 words correctly recalled out of all remember trials.

#### Letter Memory [Adapted From Morris and Jones (1990)]

Letter memory captures the ability to maintain and update items in working memory. In each trial, participants saw a series of 9, 11, or 13 consonants, with each letter appearing for 3 s, and had to say aloud the last four letters, including the current letter. The dependent measure was the proportion of 132 sets correctly rehearsed (i.e., the last four letters reported in the correct order) across 12 trials.

#### Number–Letter [Adapted From Rogers and Monsell (1995)]

Number–letter captures the ability to shift between mental sets. In each trial of the scanner version, participants saw a box sectioned into 4 quadrants. The borders of one quadrant were darkened (i.e., cued) for 350 ms, then a number–letter or letter–number pair (e.g., 4K) appeared inside until it was categorized. The participant had to categorize the number (top 2 quadrants) or letter (bottom 2 quadrants) as odd/even or consonant/vowel, respectively, using two buttons on a button box. The stimuli disappeared from the screen when categorized, and there was a 350 ms response-to-cue interval. The trials were arranged in blocks, and rest blocks (20 s) were intermixed with the task blocks. Each block was preceded by a jittered instruction (TOP, BOTTOM, MIXED, or FIXATION for 2, 4, or 6 s) that indicated where the stimuli would appear for that block. In mixed blocks, half the trials were repeat trials in which the task stayed the same as the previous trial; the other trials required a switch in categorization task. Each block consisted of 13 trials, with the first trial not counted because it was neither switch nor repeat. There were 2 runs, each containing 8 mixed blocks, 8 single-task blocks (4 each number and letter blocks), and rest blocks. The behavioral dependent measure was the local switch cost – the difference between average response times on correct switch and no-switch trials within mixed blocks (96 trials of each type).

### Category-Switch [Adapted From Mayr and Kliegl (2000)]

Category-switch captures the ability to shift between mental sets. In each trial, participants categorized a word according to animacy (i.e., living vs. non-living) or size (i.e., smaller or larger than a soccer ball), depending on a cue (heart or crossed arrows, respectively) that preceded the word by 350 ms and remained above the word until the participant responded with one of two buttons on a button box. The stimuli disappeared from the screen when categorized, and there was a 350 ms response-to-cue interval. A 200-ms buzz sounded for errors. The task began with two single-task blocks of 32 trials each, in which participants categorized words only by animacy then only by size. Then participants completed two mixed blocks of 64 trials each, in which half the trials required switching the categorization criterion. The dependent measure was the local switch cost – the difference between average response times on correct switch and no-switch trials within mixed blocks (64 trials of each type).

### T1 Structural Scan and DTI Procedure

All structural MRI data were acquired using a Siemens 3-Tesla MAGNETOM Trio MRI scanner at the University of Colorado Boulder. A 32-channel headcoil was used for radiofrequency transmission and reception. Data pertaining to gray matter structure was acquired via a T1-weighted Magnetization Prepared Gradient Echo sequence in 224 sagittal slices, with a repetition time (TR) = 2400 ms, echo time (TE) = 2.01 ms, flip angle = 8°, field of view (FoV) = 256 mm, and voxel size of 0.8 mm^3^. Diffusion-weighted data presented in this paper was acquired via a set of three scans, all with a multi-band acceleration factor of 3, capturing a total of 172 gradient directions. These scans each consisted of 72 slices, had a TR = 4000 ms, TE = 112 ms, flip angle = 84°, FoV = 224 mm, β = 3000 s/m^2^ and voxel size of 2 mm^3^, with the first and third scans captured with a phase encoding direction of left to right, and the second with a phase encoding direction of right to left.

### Data Analysis

For table describing the analysis types, steps, associated tables, and an example, see Supplementary Table S1.

#### EF Data

Scores on the six EF tasks were subjected to the same trimming and transformation used in prior studies to improve normality and reliability (Friedman et al., 2016). Specifically, RT tasks underwent within-subject trimming (Wilcox and Keselman, 2003). Though the exact number of trials that were trimmed differed between participants, on average, under 7% of trials on the Stroop and under 10% of trials on the category switch task were trimmed. Additionally, within the number–letter and category-switch tasks, RTs following error trials were excluded, as determining switch versus repeat trials is dependent on the preceding trial. Following within-subject RT trimming, extreme high and low scores at the between-subjects level (greater than ±3 *SDs* from the group mean) were replaced with the cutoff value of 3 *SD*s above or below the mean, respectively, to improve normality and reduce the impact of extreme scores while maintaining these scores in the distribution. Fewer than 3% of EF scores were adjusted by this transformation for any given task. We have used this same criterion of 3 *SD*s in prior waves of data collection with this twin sample (Friedman et al., 2016); we selected this conservative criterion because, with this large of a sample size, some cases within 3 *SDs* should be expected, and such cases have less impact on both the standard deviation of the distribution and on correlations, compared to what their influence would be in a smaller sample.

**FIGURE 1:**
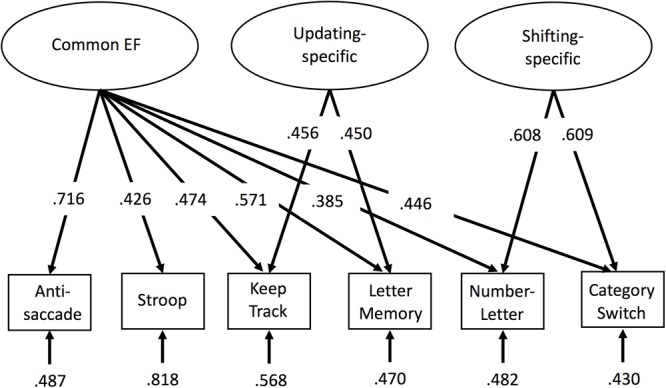
Confirmatory factor analysis model of executive functions (EFs). Rectangles represent observed EF tasks and ellipses represent latent variables. Numbers on arrows represent standardized factor loadings, and numbers at the ends of arrows represent residual variance. Common EF predicted all six EF tasks, whereas updating-specific predicted additional variance in the keep track and letter memory tasks, and shifting-specific predicted additional variance in the number–letter and category-switch tasks. All parameters were statistically significant (*p* < 0.05). EF, executive function.

Factor scores were extracted via a confirmatory factor analysis in Mplus (Muthén and Muthén, 1998), with all six EF tasks loading on common EF, the keep track and letter memory tasks loading on the orthogonal updating-specific factor, and the number–letter and category-switch tasks loading on the orthogonal shifting-specific factor (see **Figure 1**). The loadings were equated (after scaling the measures to have similar variances) within the updating-specific and shifting-specific factors to identify these two-indicator factors. These EF factor scores were then used as dependent measures for analyses of interest, including surface-based morphometry and tract based spatial statistics of diffusion data.

#### Surface-Based Morphometry

Surface-based morphometry (SBM) was carried out using the Freesurfer analysis suite^1^. We chose SBM over voxel-based techniques because SBM allows for the examination of surface area and cortical thickness in addition to volume, whereas voxel-based methods only allow for the investigation of cortical volume. Surface area and thickness are under distinct genetic control (Winkler et al., 2010), suggesting they may capture different mechanisms in neural organization that is lost by looking at volume alone. Additionally, voxel-based techniques are particularly susceptible to be confounded by partial volume effects, effects which SBM is more robust. T1-weighted structural images were brain extracted using a hybrid watershed/surface deformation procedure (Segonne et al., 2004), followed by a transformation into Talaiarch space, intensity normalization (Sled et al., 1998), tessellation of the gray/white matter boundary (Fischl et al., 2001), and surface deformation along intensity gradients to optimally differentiate gray matter, white matter, and cerebral spinal fluid boundaries (Dale et al., 1999; Fischl and Dale, 2000). The resulting segmented surfaces were registered to a standard spherical inflated brain template (Fischl et al., 1999a,b), parcelated according to gyral and sulcal structure (Fischl et al., 2004; Desikan et al., 2006), and then used to compute a range of surface-based measurements, including cortical volume, surface area, thickness, and local gyrification. Whereas cortical volume captures the total amount of gray matter within a region, and can be decomposed into two constituent parts, namely thickness and surface area, local gyrification index the degree to which the amount of surface area that is contained within the sulci of the brain.

#### Confirmatory SBM Analyses

In an attempt to replicate results found in Smolker et al. (2015), we carried out ROI analyses in which we tested for associations between mean gray matter morphometry values for each subject from the ROIs identified in Smolker et al. (2015). We then carried out multiple regression models to test for significant associations between neuroanatomy of these ROIs and EFs (see *Multiple Regression* below for details analyses).

#### Exploratory SBM Analyses

To investigate the degree to which regional variability in multiple measures of gray matter morphometry were associated with EF factor scores, we performed gray matter morphometry analyses of volume, cortical thickness, surface area, and local gyrification index via general linear models, which tested for vertex-wise associations between the aforementioned SBM measures and the EFs across the entire cortex. SBM analyses involving volume and surface area treated total intracranial volume (ICV) as a nuisance covariate, in line with recommendations from previous work (Buckner et al., 2004). Smoothing was set to a full-width-half-max parameter of 10mm, and all results that passed *p* < 0.05 where then corrected for multiple comparisons via Monte Carlo simulations (Hagler et al., 2006). These simulations generated data-driven cluster size limits for determining cluster extent significance. All reported clusters passed Monte Carlo simulations at *p* < 0.05.

#### Diffusion Tensor Imaging

Diffusion-weighted tensor images were processed with FSL (Smith et al., 2004), using the FDT toolbox (Behrens et al., 2003b, 2007) and tract-based spatial statistics (TBSS; Smith et al., 2006). All images were first motion and distortion corrected. Within each subject, diffusion tensor models were fit for each voxel, creating images of four common measures of white matter diffusion, including fractional anisotropy, mean diffusivity, radial diffusivity, and axial diffusivity, across the whole brain. Fractional anisotropy, a measure of the degree to which the motion of water molecules are constrained within neural axons, is thought to reflect the overall integrity of myelin in the brain and can be decomposed into constituent parts: including mean diffusivity, an average of the eigenvalues associated with three primary diffusion directions; axial diffusivity, the degree to which water molecules diffuse in the primary eigenvalue directions; and radial diffusivity, the average of two non-primary eigenvalues (for a review of DTI measures, see Alexander et al., 2007). For the four diffusion measures separately, all resulting subject-specific diffusion images were then non-linearly aligned to a 1mm^3^ white matter template in standard space. The aligned images were then skeletonized and averaged, creating an average skeleton mask of prominent white matter tracts. For confirmatory analyses, we calculated the mean fractional anisotropy of the white matter tracts previously implicated in emerging adults (Smolker et al., 2015): the rSLF and the bilateral inferior fontoocccipital fasciculus (iFOF). These tract-specific ROIs were defined based on the JHU white-matter tractography atlas, and values for mean FA of these white matter tracts were extracted for each subject, individually.

#### Confirmatory DTI Analyses

In an attempt to replicate results found in Smolker et al. (2015), we tested for associations between mean whole-tract fractional anisotropy of the rSLF and bilateral iFOF with common EF and shifting-specific, respectively. Because these white matter tracts may still be associated with EF, but on a more regional as opposed to whole-tract level, we carried out voxel-wise TBSS within masks of these two tracts.

#### Exploratory DTI Analyses

To investigate the degree to which multiple voxel-wise diffusion measures within major white matter tracts throughout the whole brain was associated with EF, we carried out TBSS within a skeletonized mask of prominent white matter tracts. Reported statistics for voxel-wise analyses were corrected for multiple comparisons using Threshold-Free Cluster Enhancement (TFCE) (Smith and Nichols, 2009), which provides a threshold-free method for determining significant clusters. All reported DTI clusters passed at a TFCE-corrected 1-*p* value of 0.95. For use in subsequent multiple regression analyses, we computed each subject’s mean DTI values for all clusters individually. All reported clusters passed multiple regression testing at a *p* < 0.05 after accounting for family structure and gender.

#### Cluster Selection

For all clusters that passed correction for multiple comparisons, each subjects mean neuroanatomical value across a given cluster was extracted. Because of the prevalence of twins in the current sample has the potential to inflate effect sizes by reducing sample variance, all clusters that passed correction for multiple comparisons were then corrected for family structure in the context of regressing each participants EF factor score on each participant’s mean value for a given cluster. Family structure was coded as a unique identifying number for each family, with twins receiving the same value if they belonged to the same family. These family identification numbers were then used as the grouping variable for sandwich estimation, as implemented in by MPlus’s TYPE = COMPLEX option. This procedure was performed for all clusters individually. Prior to running regression models, EF factor scores and mean neuroanatomical estimates of the identified clusters were Winsorized between-subjects, with any values above or below the 99th and 1st percentile being moved to exactly the 99th and 1st percentiles, respectively. Because of the number of distinct analyses run, it was important to adjust the alpha level for determining significance. For each of the three EF factors scores, we carried out four whole brain SBM analyses (12 test) and four whole brain DTI analyses (12 test). While we report clusters in the current manuscript that reached a standard alpha threshold of 0.05, we note that the Bonferroni corrected alpha in the current study is *p <* 0.0021. Of the 17 distinct neuroanatomical clusters identified in the current sample shown in **Table 2**, only one cluster did not pass Bonferroni level correction.

#### Multiple Regression

All multiple regression analyses were carried out using MPlus (Muthén and Muthén, 1998). We employed multiple regression with sandwich estimation to both account for family structure and interpolate effect sizes when multiple neuroanatomical predictors were used to predict a given EF factor scores. To better understand the manner in which gray matter morphometry and white matter diffusion can be used in tandem to better understand individual differences in EF, we included all neuroanatomical clusters found to be associated with a given EF, in a single model predicting that EF (i.e., the full model). This procedure enabled two important insights. First, it allowed the examination of which neuroanatomical clusters remained significantly associated with EF after taking into account all other neuroanatomical clusters associated with that EF. Distinct measures both within- and between- gray matter and white matter modalities have been shown to explain overlapping variance in individual differences in behavior (Erus et al., 2014), suggesting a potential integrative relationship across aspects of both gray matter and white matter structure that coincides with network-oriented models of the brain and cognition. Second, the full model allowed us to determine the total variance in EF factor scores that can be explained by the identified neuroanatomical clusters.

## Results

### Behavioral Results

For descriptive statistics of the six behavioral tasks used in the confirmatory factor analysis to obtain the factor scores, see **Table 1**. This confirmatory factor analysis model (see **Figure 1**) fit reasonably well, χ^2^(7) = 24.08, *p* = 0.001, CFI = 0.935, RMSEA = 0.093. Although the fit indices slightly exceeded the cutoffs typically used to indicate good fit (i.e., CFI > 0.95 and RMSEA < 0.06; Hu and Bentler, 1995), we did not implement any model modifications so as to maintain consistency with prior versions of the model that have been shown to fit well (Friedman et al., 2016). Factor score determinacy estimates for the complete data pattern were 0.83, 0.60, and 0.75 for common EF, updating-specific, and shifting-specific, respectively. Demonstrating the reliability and stability of these factor scores, common EF factor scores from this age 29 assessment correlated 0.79 and 0.68 with common EF factor scores from the 9-task batteries completed by this sample at ages 23 and 17, respectively; updating-specific factor scores from this wave correlated 0.61 and 0.44 with updating-specific scores (based on 3 tasks each) at ages 23 and 17, respectively; and shifting-specific factor scores from this wave correlated 0.62 and 0.60 with shifting-specific scores (based on 3 tasks each) at ages 23 and 17, respectively (all *p*s < 0.001).

**Table 1:** Descriptive statistics of executive function tasks.

Task	*N*	Mean (*SD*)	Range	Skewness	Kurtosis	Reliability
Antisaccade	244	43.87% (21.35)	5.00 to 96.67	0.37	–0.67	0.90^a^
Stroop	248	154.44 ms (77.34)	–3.14 to 395.60	0.81	0.67	0.96^a^
Keep Track	245	75.63% (14.12)	34.22 to 100.00	–0.66	0.03	0.74^b^
Letter Memory	251	71.48% (14.01)	35.61 to 100.00	0.06	–0.87	0.93^b^
Number- letter	243	171.12 ms (106.01)	–41.36 to 508.88	0.84	0.89	0.81^a^
Category switch	250	203.79 ms (175.29)	–64.78 to 744.85	1.33	1.49	0.94^a^

*^*a*^Reliability is split-half (odd/even for Stroop and Category-switch or run1/run2 for antisaccade and number–letter), adjusted with the Spearman–Brown prophecy formula. ^*b*^Reliability is Chronbach’s alpha across 3 runs for keep track and 4 sets of trials for letter memory. *N*, number of participants who had usable data on a given task.*

### Surface-Based Morphometry

**FIGURE 2:**
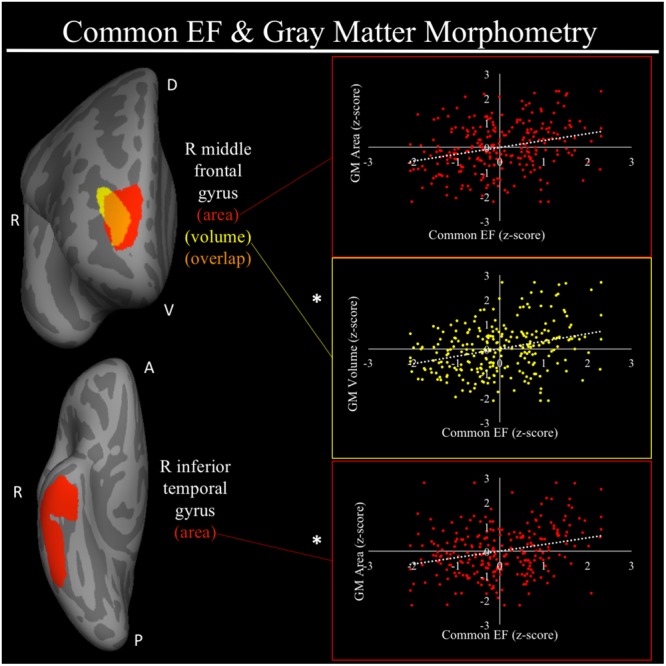
Regional gray matter clusters associated with common executive function. Cortical clusters that passed correction for multiple comparisons. All clusters remained significant after taking into account family structure. Scatterplots show simple correlation between gray matter measure total of a given ROI and common EF factor score. ^∗^Indicates clusters that remained significantly associated with common EF in the full model, in which all neuroanatomical clusters associated with cEF were included in a single model. red, greater common EF associated with greater surface area; yellow, greater common EF associated with greater volume; orange, overlap between red and yellow clusters; EF, executive function; GM, gray matter; R, right; A, anterior, P, posterior; D, dorsal; V, ventral.

**FIGURE 3:**
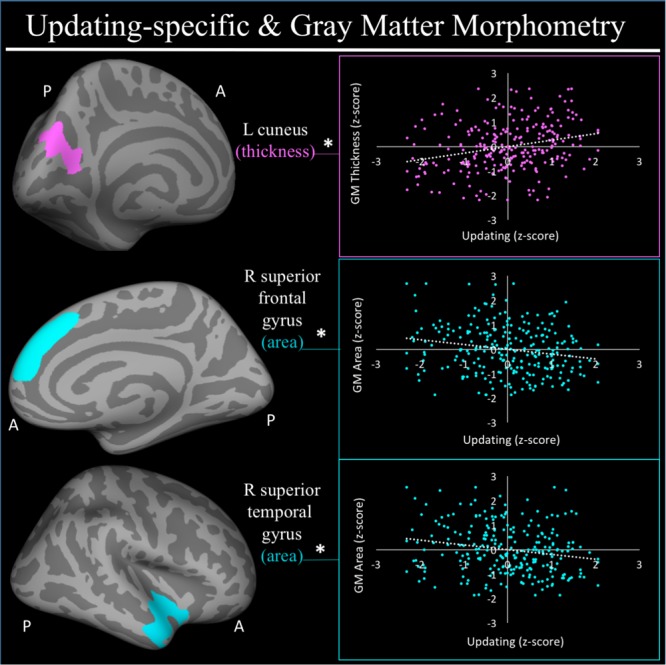
Regional gray matter clusters associated with updating-specific factor score. Cortical clusters that passed correction for multiple comparisons. All clusters remained significant after taking into account family structure. ^∗^Indicates clusters that remained significantly associated with updating-specific in the full model, in which all neuroanatomical clusters associated with updating-specific were included in a single model. Hot colors (i.e., pink) indicate greater morphometry associated with greater updating-specific factor scores. Cold colors (e.g., teal) indicated less morphometry associated with greater updating-specific ability. Scatterplots show simple correlation between morphometry total of ROI and updating-specific factor score. pink, greater updating associated greater thickness; teal, greater updating associated with less surface area; GM, gray matter; R, right; L, left; A, anterior, P, posterior.

For a full list of SBM results that passed correction for multiple comparisons and family structure, see **Table 2**. In the confirmatory ROI analyses, none of the gray matter features identified in Smolker et al. (2015) were significantly associated with EFs in the current sample. In exploratory analyses, better common EF was associated with increased volume and surface area of clusters spanning right frontal pole (FP)/right middle frontal gyrus (MFG) (volume: *x* = 24, *y* = 43, *z* = 23, *β* = 0.294, *SE* = 0.062, *p* < 0.001; area: *x* = 21, *y* = 60, *z* = 6, *β* = 0.278, *SE* = 0.069, *p* < 0.001) (**Figure 2**), as well as increased surface area of a cluster in the right inferior temporal gyrus (rITG; *x* = 47, *y* = -21, *z* = -27; *β* = 0.276, *SE* = 0.085, *p* = 0.001) (**Figure 2**). Better updating-specific was associated with increased thickness of a region of left cuneus/precuneus (lCun/PC; *x* = -21, *y* = -61, *z* = 9, *β* = 0.249, *SE* = 0.058, *p* < 0.001) (**Figure 3**), and decreased thickness of clusters in the medial portion of right superior frontal gyrus (rSFG; *x* = 8, *y* = 19, *z* = 47, *β* = -0.240, *SE* = 0.073, *p* = 0.001) and right anterior superior/middle temporal gyrus (rS/MTG; *x* = 48, *y* = 5, *z* = -27; *β* = -0.282, *SE* = 0.080, *p* < 0.001) (**Figure 3**).

**Table 2:** Significant clusters after correction for family structure.

Neuroanatomy domain	Executive function dimension	Region or tract (measure)	Cluster size (mm)	*X*	*Y*	*Z*	*β*	*SE*	*p*-value
Gray matter	cEF	rMFG/FP (vol)	407	24	43	23	0.294	0.062	<0.001
morphometry	cEF	rMFG/FP (area)	848	21	60	6	0.278	0.069	<0.001
	cEF	rITG (area)	1987	47	–21	–27	0.276	0.085	0.001
	UPD	rSFG (area)	1498	8	19	47	–0.240	0.073	0.001
	UPD	rM/STG (area)	1709	48	5	–27	–0.282	0.080	<0.001
	UPD	lCUN/PC (thick)	1160	–21	–61	9	0.249	0.058	<0.001
White matter diffusion	cEF	lATR (FA)	64	–24	15	16	0.277	0.058	<0.001
	cEF	rSLF (FA)	56	39	–9	29	0.229	0.071	0.001
	SHI	rOR (AD)	58	48	–33	–11	–0.265	0.058	<0.001
	SHI	lSLF- vent (AD)	86	–44	–13	26	–0.257	0.068	<0.001
	SHI	lSLF- dors (AD)	141	–36	–10	32	–0.209	0.067	0.002
	SHI	lSLF- post (AD)	245	–33	–34	36	–0.257	0.085	0.002
	SHI	lCC (AD)	2618	–20	–44	31	–0.260	0.073	<0.001
	SHI	whole brain (MD)	40750	–37	–25	31	–0.248	0.067	<0.001
	SHI	rOR-post (RD)	102	12	–77	22	–0.209	0.059	<0.001
	SHI	rOR-ant^!^ (RD)	186	39	–48	–15	–0.189	0.071	0.008
	SHI	whole brain (RD)	28289	–34	–36	24	–0.255	0.062	<0.001

*Clusters that passed correction for multiple comparisons and family structure. Reported stats are from regressing associated executive function dimension on a mean neuroanatomical values of a single cluster (controlling for nuisance covariates). “X”, “Y”, and “Z” represent MNI coordinates of a given cluster’s peak. ^!^ indicates that cluster’s *p*-value did not pass Bonferroni level correction of *p* < 0.0021. cEF, common executive function; UPD, updating-specific; SHI, shifting-specific; rMFG/FP, right middle frontal gyrus/frontal pole; rITG, right inferior temporal gyrus; rSFG, right superior frontal gyrus; rM/STG, right middle/superior temporal gyrus; lCUN/PC, left cuneus/precuneus cortex; lATR, left anterior thalamic radiation; lSLF, left superior longitudinal fasciculus; rSLF, right superior longitudinal fasciculus; rOR, right optic radiation; lCC, left corpus callosum; vent, ventral; dors, dorsal; post, posterior; vol, volume; thick, thickness; FA, fractional anisotropy; AD, axial diffusivity; MD, mean diffusivity; RD, radial diffusivity.*

### Diffusion Tensor Imaging

**FIGURE 4:**
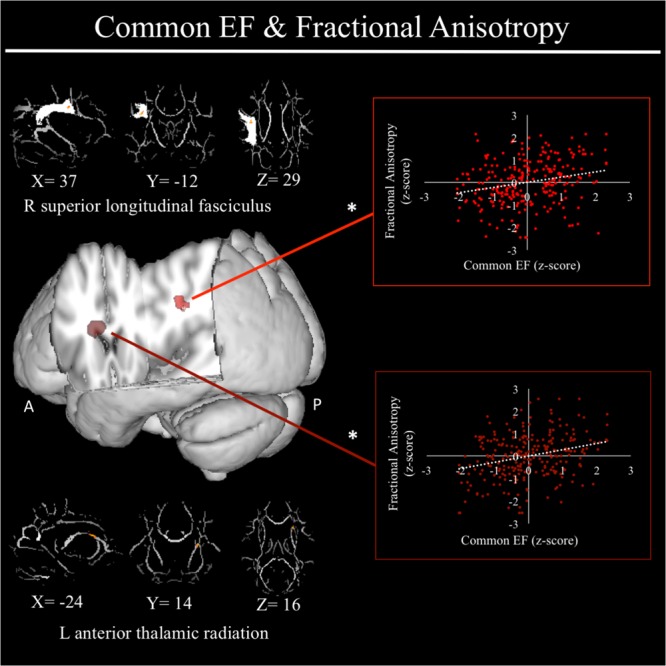
Regional fractional anisotropy clusters associated with common executive function factor scores. Significant results from Tract-Based Spatial Statistics (TBSS) within a mask of the right superior longitudinal fasciculus as well as within skeletonized mask of all major white matter tracts across the whole brain. Significant positive association were found between common EF and fractional anisotropy of a cluster in the right superior longitudinal fasciculus (shown in light red), specifically SLF-II. When conducting TBSS within skeletonized mask of all major white matter tracts across the whole brain, a significant positive association was found between common EF and fractional anisotropy of a cluster in the left anterior thalamic radiation. Scatterplots show simple correlation between fractional anisotropy total of ROI and common EF factor score. ^∗^Indicates clusters that remained significantly associated with common EF in the full model, in which all neuroanatomical clusters associated with common EF were included in a single model. R, right; A, anterior; P, posterior; X, Y, and Z are MNI coordinates of peak of cluster.

For a full list of DTI results that passed correction for multiple comparisons and family structure, see **Table 2**. Confirmatory analyses of relationships from Smolker et al. (2015) revealed that, in the current sample, better common EF was marginally associated with increased average fractional anisotropy across the entire rSLF, while shifting-specific was not significantly associated with fractional anisotropy of the bilateral iFOF. We then tested for voxel-wise associations between fractional anisotropy and EF within the white matter tract implicated in Smolker et al. (2015) finding a significant positive association of fractional anisotropy of a cluster in the anterior portion of right SLF (see **Figure 4**), specifically SLF-II, with common EF (*x* = 39, *y* = -9, *z* = 29; *β* = 0.229, *SE* = 0.071, *p* = 0.001), with greater FA associating with better common EF (**Figure 4**).

**FIGURE 5:**
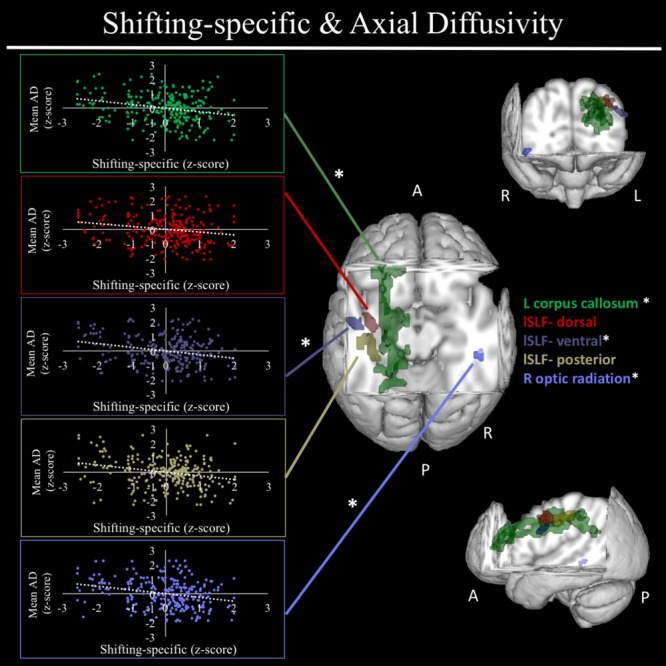
Regional axial diffusivity clusters associated with shifting-specific factor scores. Significant results from TBSS within skeletonized mask of all major white matter tracts across the whole brain. Significant negative association were found between shifting-specific factor scores and five clusters of radial diffusivity, after accounting for family structure and gender. These clusters included the left corpus callosum (shown in green), three clusters within the left superior longitudinal fasciculus (lSLF), including a more dorsal cluster (lSLF-dorsal; shown in red), a more ventral cluster (lSLF-ventral; shown in dark purple), and a more posterior cluster (lSLF-posterior) shown in light purple. Scatterplots show simple correlation between mean axial diffusivity of a given ROI and shifting-specific factor score. ^∗^Indicates clusters that remained significantly associated with shifting-specific in the full model, in which all neuroanatomical clusters associated with shifting-specific were included in a single model. R, right; L, left; A, anterior; P, posterior; X, Y, and Z are MNI coordinates of peak of cluster.

**FIGURE 6:**
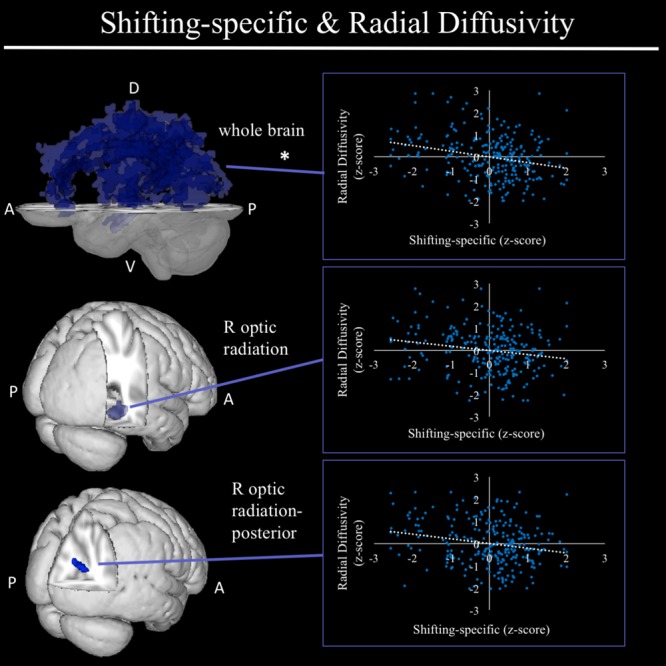
Regional radial diffusivity clusters associated with shifting-specific factor scores. Significant results from TBSS within skeletonized mask of all major white matter tracts across the whole brain. Significant negative associations were found between shifting-specific factor scores and a three radial diffusivity clusters, including a cluster spanning nearly all major white matter tracts in the whole brain (top), and two clusters within the right optic radiation, a more anterior cluster and a more posterior cluster, after accounting for family structure and gender. Of these three clusters, only the whole brain cluster remained significant in the full model, which regressed shifting-specific factor scores on all associated neuroanatomical clusters. Scatterplots show simple correlation between mean radial diffusivity of a given ROI and shifting-specific factor score. ^∗^Indicates clusters that remained significantly associated with shifting-specific in the full model, in which all neuroanatomical clusters associated with shifting-specific were included in a single model. R, right; A, anterior; P, posterior; D, dorsal; V, ventral.

**FIGURE 7:**
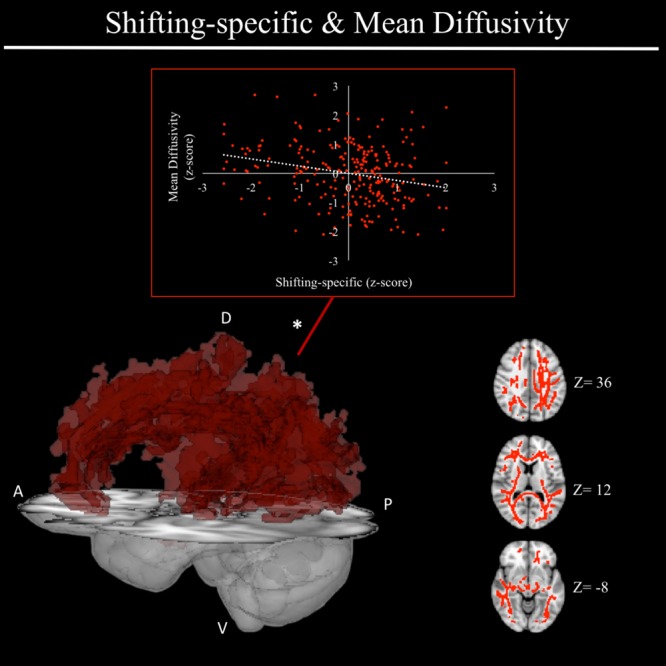
Regional mean diffusivity cluster associated with shifting-specific factor scores. Significant results from TBSS within skeletonized mask of all major white matter tracts across the whole brain. Significant positive association was found between shifting-specific factor scores and a cluster of mean diffusivity spanning nearly all major white matter tracts in the whole brain, after accounting for family structure and gender. Scatterplot shows simple correlation between average mean diffusivity across a given ROI and shifting-specific factor score. ^∗^Indicates clusters that remained significantly associated with shifting-specific in the full model, in which all neuroanatomical clusters associated with shifting-specific were included in a single model. A, anterior; P, posterior; D, dorsal; V, ventral; Z, MNI Z coordinate.

In exploratory analyses, we found a significant positive association between fractional anisotropy in a cluster of the left anterior thalamic radiation (lATR; *x* = -24, *y* = 15, *z* = 16; *β* = 0.277, *SE* = 0.058, *p* < 0.001) and common EF (**Figure 4**) with better common EF associated with greater fractional anisotropy. No significant DTI results were found for updating-specific, though we found a number of DTI clusters that were significantly associated with shifting-specific. Clusters in which DTI properties were associated with shifting-specific included axial diffusivity of a clusters in the right optic radiation (rOR; *x* = 48, *y* = -33, *z* = -11; *β* = -0.265, *SE* = 0.058, *p* < 0.001) (**Figure 5**), three clusters within the left SLF (**Figure 5**), including a more ventral cluster (lSLF-vent; *x* = -44, *y* = -13, *z* = 26; *β* = -0.257, *SE* = 0.068, *p* < 0.001), a more dorsal cluster (lSLF-dors; *x* = -36, *y* = -10, *z* = 32; *β* = -0.209, *SE* = 0.067, *p* = 0.002), and a more posterior cluster (lSLF-post; *x* = -33, *y* = -34, *z* = 36; *β* = -0.257, *SE* = 0.085, *p* = 0.002), and a cluster spanning almost the entirety of the left corpus callosum (lCC; *x* = -20, *y* = -44, *z* = 31; *β* = -0.260, *SE* = 0.073, *p* < 0.001) (**Figure 5**). Shifting-specific was also associated with radial diffusivity in two clusters in the rOR (**Figure 6**) – a more anterior cluster (rOR-ant; *x* = 39, *y* = -48, *z* = -15; *β* = -0.189, *SE* = 0.071, *p* = 0.008) and a more posterior cluster (rOR-post; *x* = 12, *y* = -77, *z* = 22; *β* = -0.209, *SE* = 0.059, *p* < 0.001) – as well as radial diffusivity of a cluster spanning much of the brain (whole brain; *x* = -34, *y* = -36, *z* = 24; *β* = -0.255, *SE* = 0.062, *p* < 0.001) (**Figure 6**) as well an mean diffusivity of a cluster spanning much of the brain (whole brain; *x* = -37, *y* = -25, *z* = 31; *β* = -0.248, *SE* = 0.067, *p* < 0.001) (**Figure 7**).

### Cross- and Within-Modality Multiple Regression

For a full list of clusters that remained significantly associated with a given EF when included in a model with other clusters associated with that EF, see **Table 3**. The only EF dimensions for which we observed both significant gray matter and DTI predictors was common EF, resulting in a model including volume of rFP/MFG cluster (*β* = 0.222, *SE* = 0.112, *p* = 0.048), area of the rITG cluster (*β* = 0.292, *SE* = 0.076, *p* < 0.001), FA of the rSLF cluster (*β* = 0.164, *SE* = 0.068, *p* = 0.015), and fractional anisotropy of the lATR cluster (*β* = 0.225, *SE* = 0.058, *p* < 0.001) as predictors of common EF, all of which remained significantly positively associated with common EF (full model *R^2^*= 0.225, *SE* = 0.046, *p* < 0.001) (**Table 3**). When all significant gray matter clusters associated with updating-specific were included in a single model controlling for family structure, total ICV, and gender, the lCun/PC cluster (*β* = 0.214, *SE* = 0.059, *p* < 0.001), rSFG cluster (*β* = -0.152, *SE* = 0.070, *p* = 0.030), and rS/MTG clusters (*β* = -0.180, *SE* = 0.077, *p* = 0.020) all remained significantly associated with updating-specific (full model *R^2^*= 0.179, *SE* = 0.051, *p* < 0.001) (**Table 3**). When all significant DTI clusters associated with shifting-specific were included in a single model controlling for family structure and gender (**Table 3**), the axial diffusivity cluster in rOR (*β* = -0.200, *SE* = 0.054, *p* < 0.001), the axial diffusivity cluster lSLF-vent (*β* = -0.173, *SE* = 0.057, *p* = 0.002), the axial diffusivity cluster in lCC (*β* = -0.357, *SE* = 0.110, *p* = 0.001), the whole brain mean diffusivity cluster (*β* = 1.201, *SE* = 0.242, *p* < 0.001), and the whole brain radial diffusivity cluster (*β* = -0.933, *SE* = 0.205, *p* < 0.001) cluster remained significantly associated with updating-specific (full model *R^2^*= 0.237, *SE* = 0.051, *p* < 0.001).

**Table 3:** Results from cross- and within- modality multiple regression.

Behavioral dimension	Region or tract (measure)	*β*	*SE*	*p*-value
cEF	rMFG/FP (vol)	0.222	0.112	0.048
	rITG (area)	0.292	0.076	<0.001
	rSLF (FA)	0.164	0.068	0.015
	lATR (FA)	0.225	0.058	<0.001
UPD	rSFG (area)	–0.152	0.070	0.030
	rM/STG (area)	–0.180	0.077	0.020
	lCUN/PC (thick)	0.214	0.059	<0.001
SHI	rOR (AD)	–0.200	0.054	<0.001
	lSLF-vent (AD)	–0.173	0.057	0.002
	lCC (AD)	–0.357	0.110	0.001
	whole brain (MD)	1.201	0.242	<0.001
	whole brain (RD)	–0.933	0.205	<0.001

*Significant results from “final model,” in which executive function factor scores were regressed on all gray matter morphometry and diffusion tensor imaging (DTI) clusters that remained significant after accounting for family structure, simultaneously, in a single model. The only executive function factor score for which both gray matter morphometry and DTI clusters were found was common executive function (cEF). For updating-specific (UPD) and shifting-specific (SHI), displayed results are from models including all associated gray matter (updating-specific) and DTI (shifting-specific) clusters, respectively. rFP/MFG, right frontal pole/middle frontal gyrus; rITG, right inferior temporal gyrus; rSFG, right superior frontal gyrus; rM/STG, right middle/superior temporal gyrus; lCUN/PC, left cuneus/precuneus cortex; lATR, left anterior thalamic radiation; rSLF, right superior longitudinal fasciculus; rOR, right optic radiation; lSLF-vent, left superior longitudinal fasciculus – ventral; lCC, left corpus callosum; vol, volume; thick, thickness; FA, fractional anisotropy.*

## Discussion

The current study tested for associations between neuroanatomical measures and the three distinct EF constructs of the unity/diversity model of EF in a non-clinical sample closely clustered around 29 years of age. We observed relationships between common EF and multiple gray matter and fractional anisotropy characteristics. Updating-specific was associated with gray matter properties only, while shifting-specific was associated with a range of properties of white matter, including regional variability in mean, radial, and axial diffusivity. It is important to note that, while the effect sizes of neuroanatomy-EF relationships observed in the current study may be considered weak, it is unlikely that large portions of variance in complex cognitive behaviors in healthy individuals will be explained by neuroanatomy alone. Instead, neuroanatomy represents one piece of what are likely highly complex, multimodal brain systems supporting complex behaviors. We center our discussion around two questions: (1) whether the areas of gray matter and white matter that show associations with EF are within or outside the FPN; and (2) whether the pattern of results observed is consistent with what we have observed previously in a sample of emerging adults.

### Neuroanatomical Correlates of EF: Within or Outside the FPN?

We observed that the gray matter morphometry of regions that are associated with common EF and updating-specific did not fall squarely within the FPN, but instead fell in brain regions commonly associated with default mode network (DMN), as well as regions supporting non-EF processes vital to task performance. The majority of the observed associations were in regions outside of the FPN, with only two associations occurring with clusters that spanned the FPN. Moreover, we observed that decreased gray matter in regions commonly associated with DMN were associated with better updating-specific. The DMN has been shown to have inverse associations with the efficacy of FPN engagement (Fox et al., 2005; Elton and Gao, 2015), as well as regions supporting non-EF processes vital to task performance. In terms of white matter, results suggested that individuals with higher EF are characterized by the properties of white matter tracts connecting a range of brain regions, including prefrontal to more posterior brain regions, as inferred from DTI measures.

One of the two major results regarding gray matter properties and common EF was an association between increased volume and surface area of the rFP/MFG and higher common EF. Comparing the spatial location of these clusters to a popular seven-network parcellation of brain networks (Yeo et al., 2011) the more lateral aspects of the rFP/MFG clusters lie in cortex associated with the FPN, whereas the more medial portions of these clusters lie in cortex associated with the DMN. Hence, the region so-identified does not fall squarely within mid-dlPFC region that has been suggested to be at the top of a neuroanatomical hierarchy for EF (Nee and D’ Esposito, 2016, 2017), but rather it is located a bit more dorsal and anterior. The FP has been implicated by our group and others with high-level goal representations (Gilbert et al., 2006; Burgess et al., 2007, 2008; Tsujimoto et al., 2011; Orr and Banich, 2014; Orr et al., 2015). For example, Orr and Banich (2014) found there is greater FP activation when task goals must be voluntarily selected by an individual as compared to when they are given explicit instructions regarding the task goal. This finding is consistent with models of FP function as biasing behavior in accordance with internal goals in the absence of external goal cues (Burgess et al., 2007). Evidence from DTI and functional co-activation suggests that dorsal portions of the FP, with which the common EF cluster in the current study is contiguous, have short-range projections to other PFC regions. Such projections may allow for the updating of goal-related information in more mid-dorsolateral regions, which then in turn, can modulate activity of posterior regions in accordance with task goals (Orr et al., 2015). Thus, it may be that the structural characteristics of the FP associated with higher common EF may influence the processing of higher-level goal representations. In line with this idea, common EF has been theorized to capture the maintenance of goal information that is used to bias lower-level processing in pursuit of these goals (Friedman and Miyake, 2017).

The second major gray matter result for common EF was that higher cEF was associated with greater surface area along the ventral surface of anterior right ITG, (Ishai et al., 1999; Visser et al., 2010, 2012; Peelen and Caramazza, 2012), which is not part of what is commonly considered the FPN. Rather, anterior ITG has been linked to conceptual information regarding a visual object, including semantic information, location, and associated action (Visser et al., 2010, 2012; Peelen and Caramazza, 2012). We may have found this association because the majority of EF tasks that load on the cEF factor require the interpretation of visual cues. For example, during the category-switch task, participants are presented with two cues that have distinct semantic judgments associated with them and must rapidly identify the visual cue and access the appropriate semantic category, a function which has been ascribed to anterior ITG (Visser et al., 2010, 2012). In the antisaccade and number–letter tasks, participants must identify the location of visual cues and use this location information to inform subsequent actions, once again, a function ascribed to anterior ITG (Peelen and Caramazza, 2012). The involvement of access to higher-order conceptual information pertaining to visual objects, likely grounded in anterior ITG function, may be a prerequisite for good performance on nearly all visually based EF tasks. Given that common EF captures mechanisms involved across all EF tasks, it is not surprising the brain regions supporting the conceptual information of visual objects show associations with common EF. It should be noted that such a finding does not necessarily suggest that this association is an “artifact” of using visual tasks to assess EF. Rather, it may suggest that individuals with higher common EF abilities may be better able to process information regarding lower-level processing in the context of current task goals.

With regards to white matter, higher common EF was associated with increased fractional anisotropy of white matter in clusters of the rSLF and the lATR. The SLF is often considered to be a key anatomical connection connecting frontal and parietal regions of the FPN, and has been implicated in higher-level cognitive processes, including selective attention, working memory, and EF (Vestergaard et al., 2011; Smolker et al., 2015; Urger et al., 2015). Of the five primary subcomponents that make up the SLF (Kamali et al., 2014), the cluster associated with common EF lay in the SLF-II subcomponent, which has been shown to connect the angular gyrus to middle frontal and precentral gyri (Wang et al., 2016). Though the SLF-II likely plays a role in a wide range of functions, it has been suggested to be preferentially associated with the regulation of spatial attention, with some suggesting that it plays a critical role integrating the dorsal- and ventral- attention networks, mediating information flow related to goal-directed attention (originating from dorsal attention network via SLF-I) and attention to salient events (originating from ventral attention network via SLF-III) (De Schotten et al., 2011). In the context of common EF, this purported function of integrating goal-oriented attentional signals with automatic, salient spatial attention to objects is likely involved in all, if not the majority of EF tasks. That is, all of the EF tasks paradigms that went into the common EF factor score required participants to guide spatial attention in accordance with task goals, and the ability to successfully do this is likely contingent upon the properties of the neural systems supporting spatial attention, including the SLF and its subcomponents. As such, the white matter findings are consistent with those regarding gray matter as both point to the possibility that individuals with higher common EF have associations with aspects of brain neuroanatomy that would be suggestive of expanded involvement of both top–down and bottom–up brain regions as well as their integration.

The lATR, the other white matter tract associated with common EF, connects the mediodorsal nucleus of the thalamus to the PFC (Behrens et al., 2003a; Jang and Yeo, 2014). Showing three distinct functional connectivity profiles, the mediodorsal nucleus has been shown to have dissociable connections with orbitofrontal cortex, ventrolateral PFC, and dlPFC, all of which pass through the ATR (Jang and Yeo, 2014). Indeed, in the current sample, *post hoc* analyses revealed a significant positive correlation between fractional anisotropy of the lATR cluster and volume of the right FP/MFG cluster *(r =* 0.238, *p* < 0.001), suggesting a potential neural circuit important to common EF ability, though each cluster appeared to predict unique portions of variance in common EF. Despite few if any studies implicating fractional anisotropy of the ATR in individual differences in EF amongst healthy young adults, fractional anisotropy of the ATR has been shown to be reduced in patient populations, with the degree of reduction associating with EF impairment (Mamah et al., 2010). Moreover, the mediodorsal nucleus of the thalamus has been proposed to play an important role in rapid learning of an associative nature as well as decision-making paradigms that involve multiple cognitive processes (Mitchell, 2015), exactly the type of processes tapped by the EF tasks in our behavioral battery. Hence, individuals with higher common EF have increased fractional anisotropy, often taken as an index of structural integrity (Alba-Ferrara and de Erausquin, 2013), of both a tract that connects cortical regions to prefrontal cortex (i.e., rSLF) and well as a tract that connects subcortical regions to prefrontal cortex (i.e., lATR).

With regards to updating-specific, four major associations with gray matter morphometry were observed. One of these was an association between better updating-specific and decreased surface area of the rSFG in a cluster spanning cortex both FPN and DMN. This rSFG cluster spans the dorsal portions of both the middle and anterior zones of the medial frontal cortex identified in a recent meta-analytic parcellation by de la Vega et al. (2016), although the majority of it falls within the anterior zone. The dorsal portion of the middle zone is associated with working memory and cognitive control de la Vega et al., 2016) and shows high degrees of co-activation with key components of the FPN. While this posterior portion of the rSFG cluster falls within this middle zone attributed to the FPN, the majority of this cluster sits in a region of medial PFC commonly attributed to the DMN. This portion of the anterior zone has been strongly implicated with social processing, including social perception and self-referential thought (Mitchell et al., 2005; de la Vega et al., 2016). Though it is unclear how social perception and self-referential thought relate to updating-specific or the functional consequences of reductions in surface area are, one possibility is that better updating-specific is associated with reduced engagement of these inwardly directed modes of thought. In line with this interpretation, we also observed that better updating-specific is associated with reduced surface area of the anterior right M/STG, a region implicated as in the DMN (Yeo et al., 2011), as well as affective processing (Olson et al., 2007).

In contrast, updating-specific was associated with increased cortical thickness of a region that spanned from dorsal regions of the left cuneus/precuneus, commonly implicated in visual attention (Vanni et al., 2001), to more ventral regions reaching the posterior cingulate. Though not a classic EF region *per se*, the cuneus/precuneus is frequently implicated in EF tasks due to a reliance on rapid visual processing (Wager and Smith, 2003; Simões-Franklin et al., 2010). For proper updating to occur, the environment must be monitored for cues indicating an update is needed and, in the case of the EF tasks in this study, these cues can only be discerned through rapid visual processing, to which the cuneus is a key contributor. The posterior cingulate region is one of the core hubs of the DMN, and becomes active when individuals make self-relevant, affective decisions (Andrews-Hanna et al., 2010). Like the region in the rSFG, this region spanned areas typically considered to be both the FPN and the DMN. Whether this association is indicative of alterations in individuals with higher updating-specific ability in the interaction between these two systems, which commonly activate in an antagonistic manner (Fox et al., 2005), remains to be seen and will require examination of functional patterns of brain activation.

Though no significant associations between gray matter morphometry and shifting-specific were observed in the current sample, quite a number of associations were found between regional variability in the white matter diffusion measures and individual differences in shifting-specific factor scores. One potential interpretation of shifting-specific’s exclusive associations with white matter properties may be that shifting is reliant on more transient neural processes, such as the ability to effectively reconfigure task sets and representations, and to quickly clear or replace no-longer relevant representations (Herd et al., 2014). Such reconfiguration may be dependent upon the efficiency of connectivity between multiple brain regions, with connectivity largely driven by diffusion properties of white matter (Skudlarski et al., 2008), not regional gray matter morphometry.

Specifically, we found shifting-specific ability to be associated with multiple white matter characteristics including axial diffusivity of three clusters of the lSLF, axial diffusivity of the left corpus callosum, axial diffusivity of a portion of the rOR, mean diffusivity of a cluster spanning much of the entire brain, radial diffusivity of a similar cluster spanning much of the brain, as well as radial diffusivity of two clusters within the rOR. When viewed as a whole, these results suggest three general points regarding the diffusion correlates of shifting-specific. First, the whole brain clusters identified in both mean diffusivity and radial diffusivity analyses suggest that shifting-specific is associated with diffusion properties across the entire brain. Though these results were not expected, they suggest that shifting-specific ability is at least partially dependent upon general white matter properties not just those linked to specific portions of discrete tracts. Interestingly, despite the considerable spatial overlap between the regions identified as associated with mean diffusivity and radial diffusivity, respectively and the fact that radial diffusivity is a mathematical component of mean diffusivity (Alexander et al., 2007), both clusters remained significantly associated with shifting-specific, even after taking into account the other cluster. This finding suggests that, while highly associated, mean diffusivity and radial diffusivity have distinct associations with behavior that are not captured by one measure alone, providing credence to methodologies that aim to investigate multiple measures of white matter diffusion in tandem.

A second point from the DTI analyses of shifting-specific is that, whereas cEF has been shown to be associated with diffusion properties of the *right* SLF in emerging adults (Smolker et al., 2015) and in the current sample, shifting-specific ability appears to be related to regional axial diffusivity of clusters in the *left* SLF, specifically SLF-II. As discussed previously, the SLF, particularly SLF-II, allows for long-range connections between the prefrontal and parietal cortices, including regions implicated in the FPN, and has been implicated in the regulation of attention, along with other EF-associated behaviors (Vestergaard et al., 2011; Smolker et al., 2015; Urger et al., 2015). The observed left lateralization of this relationship between shifting-specific and axial diffusivity of the SLF may reflect the linguistic nature of the shifting tasks, as the lSLF (Maldonado et al., 2011; Urger et al., 2015) and left hemisphere in general (Binder et al., 1995), have been heavily implicated in linguistic and semantic processing. We additionally found that axial diffusivity of a cluster spanning most of left hemisphere portions of the corpus callosum was negatively correlated with shifting-specific, such that better shifting-specific was associated with reduced axial diffusivity in these regions. Unlike the majority of white matter tracts that generally run anteriorly to posteriorly, the corpus callosum is the main anatomical pathway connecting the two hemispheres (Roland et al., 2017), but also has vertical projections which innervate the major lobes of the brain (Hofer and Frahm, 2006). The lSLF clusters found to be associated with shifting-specific lay directly adjacent to the more anterior section of the corpus callosum cluster, that connect to prefrontal regions. This finding raises the possibility that these clusters are capturing distinct portions of an integrated neural circuit important for determining individual differences in shifting-specific ability. In fact, prior work has shown that lower switch costs in individuals are associated with greater coupling of right and left MFG activity and that such coupling is predicted by greater volume of anterior regions of the corpus callosum (Baniqued et al., 2018). Such findings are also consistent with the notion that engaging both hemispheres is particularly helpful to task performance under conditions of higher level demand (Banich, 1998), which well describes EF tasks.

The third notable result with shifting-specific was the considerable evidence implicating distinct portions of the rOR in shifting-specific ability. Specifically, shifting-specific was found to be associated with clusters of axial and radial diffusivity in two adjacent portions of the optic radiation, as well as a second radial diffusivity cluster where the optic radiation terminates in the medial occipital lobe. Though the axial diffusivity optic radiation cluster was the only cluster of these three to remain significant after taking into account all other DTI clusters associated with shifting-specific, the fact that we found associations between shifting-specific and multiple portions of the optic radiation, across multiple diffusion measures, provides converging evidence for this relationship. Likely serving a supportive role to EF mechanisms, the optic radiation, which spans from the lateral geniculate nucleus to the occipital cortex (Yamamoto et al., 2007) provides a pathway for visual information to travel from the retina to the primary visual cortex. It is not entirely surprising to find associations between behavioral measures grounded in visual tasks to be associated with properties of the optic radiation, properties which presumably may influence the rate and efficacy with which visual information enters the visual cortex. Why a relationship would be observed with shifting-specific and not the more general common EF factor remains unclear and a potential point of further inquiry.

Given the patterns observed, it is important to consider potential explanations for associations between individual differences in EF with neuroanatomical properties outside of the classic brain regions thought to support EF. First, as discussed above, the non-FPN regions associated with individual differences in EF might indicate that individuals with higher EF rely on a larger or more diverse set of brain regions than those with lower EF. In other words, individuals who have higher EF may employ additional brain systems while performing EF tasks that are not engaged by individuals who have lower EF, or vice versa. Second, individuals with higher EF may have distinct anatomical characteristics of regions that often are observed to work in opposition to the FPN. The results indicated that better updating-specific was associated with reduced area in two regions of the brain, the superior medial frontal cortex and anterior sections of the right superior/middle temporal gyrus, that are associated with the DMN. Research has shown that activity in these two networks are often anti-correlated (Fox et al., 2005). Of course, it is impossible to determine patterns of activation on the basis of neuroanatomy, so for now these ideas are mainly speculative and will need to be evaluated by investigations focused on individual differences in EF and brain activation.

### Comparing Current Results to Younger Individuals

Another lens through which to interpret the results of this study is in comparison to our previous examination (Smolker et al., 2015) in which we similarly investigated the relationship between neuroanatomy and these same aspects of EF (common EF, updating-specific, shifting-specific). That study varied from the present one in two ways. First, it was performed on a younger sample of college-aged individual, who can be considered emerging adults. Second, we used factor scores based on a battery of three EF tasks (antisaccade, category-switch, and keep-track) rather than six, as in the present study. As in the current study, Smolker et al. (2015) found that common EF was associated with white matter tracts that connect to prefrontal and posterior regions, namely the rSLF. While the relationship with the entire rSLF was only marginal in the current sample, when we ran voxel-wise FA analyses within a mask of the rSLF, we found a significant positive association between a portion of SLF-II and common EF, suggesting that the rSLF is important to individual differences in common EF across both emerging and young adulthood. In addition, as in our prior study most of the associations observed with EF in the current study occurred in brain regions outside of the FPN.

However, the specific gray matter regions implicated and their general directionality for the most part differed between the two studies. Whereas reduced volume in bilateral ventromedial PFC was associated with better cEF in emerging adults, the current study found increased rFP/MFG volume to be associated with better cEF performance in our young adult sample. In the emerging adult sample, better updating-specific was associated with reductions in gray matter volume in left dlPFC, while the current study found increased updating-specific associated with reductions in surface area of a medial cluster of the right SFG, reductions in surface area of a cluster in right anterior temporal lobe, and increases in thickness of a cluster spanning cuneus/precuneus. The current study did not find any significant associations between shifting-specific ability and regional gray matter morphometry, whereas in our prior study there was an association between better shifting-specific ability and reduced gray matter volume in left ventrolateral PFC (BA 10/47). Additionally, whereas Smolker et al. (2015) found associations between shifting-specific and mean fractional anisotropy of the inferior frontooccipital fasciculus, in the current sample shifting-specific was not associated with FA anywhere in the brain, and instead was associated with mean diffusivity, radial diffusivity, and axial diffusivity within a number of regional clusters.

Though no formal tests were carried out comparing the current sample with the younger sample in Smolker et al. (2015), we speculate that the discrepancies between these two studies may emerge from differences in the age of the participants. At a mean age near 29 years, the current study employed a sample which is almost a decade older on average than the sample used in Smolker et al. (2015). By age 30 or so, aspects neurodevelopment, particularly of the PFC, have likely stabilized (Sowell et al., 2003), whereas neurodevelopment was likely still on-going in the younger sample (Smolker et al., 2015). Supporting this conjecture, we found that reductions in volume were associated with EF in Smolker et al. (2015), suggestive that greater developmental pruning is associated with better EF. In that sample we also found that local gryification index was a potent predictor of individual differences in EF, but observed no relationships with local gyrification index in the current study. Local gyrification index has been found to show reductions during the late teens/early 20s (Klein et al., 2014), likely driven by increases in underlying white matter characteristics (Ribeiro et al., 2013). This pattern also suggests that on-going developmental processes may be influencing associations with EF in this younger sample. Such findings are consistent with prior studies indicating that neurodevelopment has profound effects on the brain regions utilized for specific cognitive functions (Rubia et al., 2000). Nonetheless, the current study coupled with Smolker et al. (2015) do not provide a clear trajectory of how the neuroanatomical characteristics associated with EF change during the 20s. Large-scale longitudinal studies will be needed to investigate the dynamic evolution of the neural systems associated with EF performance.

### Limitations and Future Directions

The current study is not without limitations. First, a limitation to the current study is that analyses were carried out in a univariate fashion, despite evidence that behaviorally relevant neuroanatomical properties segregate into multivariate components (Xu et al., 2009a,b; Brown et al., 2012). Second, the age range of our participants is rather restricted, which may bring about reduced variability in neuroanatomy between subjects. On the other hand, having such a large sample in this relatively narrow age range, provided a clear picture of the associations between brain anatomy and EF during young adulthood. An additional limitation is that, without testing in a replication sample, it is unclear if the current results reflect biologically real associations or chance variation that can influence such studies. Finally, despite having a sample of twins and more power than most neuroimaging studies, we are currently underpowered for twin models. Following the completion of data collection for the larger study of which this project is a part, we plan to investigate (1) the replicability of the current findings in a well-matched replication sample, and (2) the degree to which neuroanatomical correlates of individual differences in EF are driven by genetic or environmental factors.

## Conclusion

Within a sample of developmentally mature young adults, common EF and updating-specific were associated with distinct properties of regional gray matter morphometry and the location of these features fell both within and outside of the FPN. Additionally, common EF was associated with fractional anisotropy of clusters in the rSLF and lATR while shifting-specific was associated with diffusion properties of multiple white matter tracts throughout the brain. These results suggest that individual differences in EF are associated with properties of neural systems of not only brain regions classically thought to support EF, but also brain systems associated with processes not traditionally conceptualized as supporting EF. These latter regions fall into one of two categories: those that are likely to support higher-order, amodal cognitive processes (e.g., goal maintenance, semantic processing) or those that allow for improved categorization of relevant perceptual information (e.g., visual processing and attentional control areas), both of which could aid performance during complex EF tasks. Coupled with the white matter findings, these results suggest that individuals with higher EF may have a more expanded, integrated and/or connected neural substrate associated with EF performance, a hypothesis that should be tested further by multimodal follow up studies. The current findings show distinct patterns of neuroanatomy-EF associations from what we have observed in younger individuals (Smolker et al., 2015), suggesting that the significant development of cortical organization occurring well into the third decade of life influence the underlying neuroanatomical characteristics associated with EF.

## Author Contributions

JH and NF designed the study that collected the data. HS, MB, and NF conceived the current analysis plan. HS and NF carried out statistical analyses included in this manuscript. HS, NF, MB, and JH wrote, revised, and/or provided comments throughout the writing of the manuscript.

## Conflict of Interest Statement

The authors declare that the research was conducted in the absence of any commercial or financial relationships that could be construed as a potential conflict of interest.

## Footnotes

1 https://surfer.nmr.mgh.harvard.edu/
