# Supplementary material for: Neuroanatomical Correlates of the Unity and Diversity Model of Executive Function in Young Adults
Source: Front Hum Neurosci. 2018 Jul 20;12:283. doi: 10.3389/fnhum.2018.00283 (PMC6064948; doi:10.3389/fnhum.2018.00283)
Supplement: Supplementary file 1 [file Table_1.DOCX]

| **Analysis Type** | **Modality**  **(measure)** | **Procedure** | **Example Analyses on Common EF** |
| --- | --- | --- | --- |
| Confirmatory | SBM  (vol./LGI) | **Direct Confirmation**   1. For each subject, extract mean GM measure within GM ROIs found in Smolker et al., 2015. 2. Regress EF factors scores on GM value of ROIs, following GM/EF relationships observed in Smolker et al., 2015 | 1. For each subject, extract mean volume and LGI from the PFC cluster found to be associated with common EF (cEF) in Smolker et al., 2015. 2. Regress cEF on mean volume and LGI of this cluster, separately, in two distinct models |
|  | DTI  (FA) | **Direct Confirmation**   1. For each subject, extract mean FA values for WM tracts found in Smolker et al., 2015. 2. Regress EF factors scores on mean FA value for ROIs, following FA/EF relationships observed in Smolker et al., 2015, accounting for family structure | 1. For each subject in the current sample, extract mean FA from the white matter tract (rSLF) found to be associated with cEF in Smolker et al., 2015. 2. Regress cEF on mean FA of the rSLF. |
|  |  | **Extension**   1. Run FA TBSS on EF within masks of WM tracts found in Smolker et al., 2015., testing the FA/EF relationships observed previously 2. For each subject, extract mean FA from each cluster observed in step 1. 3. Regress EF factors scores on mean associated FA values of clusters from steps 1 and 2. **(for significant results, see Table 2, Fig. 4)** | 1. Run FA TBSS on cEF within masks of rSLF. 2. For each subject, extract mean FA from clusters observed in step 1. 3. Regress cEF factors scores on mean FA values of rSLF cluster derived in step 2 |
| Exploratory | SBM  (all) | **Cluster Identification and Family Structure Correction**   1. Test for vertex-wise associations between GM morphometry measures and EF factor scores. 2. Correct for multiple comparisons 3. For each subject, extract mean GM morphometry measures of any clusters that passed correction for multiple comparisons carried out in step 2. 4. For each cluster identified in step 2, individually, regress associated EF factors score on mean GM morphometry value for a given cluster, accounting for family structure **(for significant results, see Table 2, Figs.4-7)**. | 1. Test for vertex-wise associations between GM morphometry measures and cEF factor scores. 2. Correct for multiple comparisons 3. For each subject, extract mean area and volume from the rFP/MFG clusters, and area of rITG cluster. 4. For the three clusters mentioned above separately, run regression predicting cEF from a given cluster, correcting for family structure, total ICV, and gender. |
|  | DTI  (all) | **Cluster Identification and Family Structure Correction**   1. Using TBSS, test for voxel-wise associations between DTI measures and EF factor scores. 2. Correct for multiple comparisons 3. For each subject, extract mean DTI measure from any clusters that passed correction for multiple comparisons carried out in step 2. 4. For each cluster identified in step 2, individually, regress EF factor scores associated with that cluster on mean DTI measure of that cluster **(for significant results, see Table 2, Figs. 4-7)** | 1. Test for voxel-wise associations between DTI measures and cEF factor scores. 2. Correct for multiple comparisons 3. For each subject, extract mean FA from lATR cluster. 4. Run regression predicting cEF from lATR, correcting for family structure and gender. |
|  | SBM  &  DTI | **Cross- and Within- Modality Regression**   1. Regress EF factor scores on all associated GM and DTI clusters that remained significant after step 5 of exploratory GM and DTI analyses outlined above, in a single model **(for significant results, see Table 3, results with * in Figs. 4-7)** | 1. Predict cEF factor scores from mean volume of rFP/MFG cluster, mean area of rITG cluster, mean FA of rSLF cluster, and mean FA of lATR cluster, in a single model. |

**Table S1: Step-by-step analysis plan.** Table showing the discrete analysis steps taken. Includes information pertaining to “Analysis Type” (confirmatory/exploratory), neuroanatomical modality and measures employed, procedure of analysis, and an example based off the results found for common EF. EF= executive function; SBM= surface-based morphometry; DTI= diffusion tensor imaging; FA= fractional anisotropy; GM= gray matter; vol.= volume; LGI= local gyrification index; PFC= prefrontal cortex; rSLF= right superior longitudinal fasciculus; rFP/MFG= right frontal pole/middle frontal gyrus; lATR= left anterior thalamic radiation.
